# Electrochemical hydrogenation of enones using a proton-exchange membrane reactor: selectivity and utility

**DOI:** 10.3762/bjoc.18.107

**Published:** 2022-08-19

**Authors:** Koichi Mitsudo, Haruka Inoue, Yuta Niki, Eisuke Sato, Seiji Suga

**Affiliations:** 1 Division of Applied Chemistry, Graduate School of Natural Science and Technology, Okayama University, 3-1-1 Tsushima-naka, Kita-ku, Okayama 700-8530, Japan

**Keywords:** enone, hydrogenation, iridium, palladium, PEM reactor

## Abstract

Electrochemical hydrogenation of enones using a proton-exchange membrane reactor is described. The reduction of enones proceeded smoothly under mild conditions to afford ketones or alcohols. The reaction occurred chemoselectively with the use of different cathode catalysts (Pd/C or Ir/C).

## Introduction

Catalytic hydrogenation of α,β-enones is a significant transformation in organic synthesis [1]. Hydrogenation of enones can give ketones, allyl alcohols, and saturated alcohols, and the control of the chemoselectivity is important. Therefore, there have been numerous studies on the hydrogenation of enones using homogeneous and heterogeneous catalysts.

Meanwhile, electrochemical systems using a proton-exchange membrane (PEM) reactor have been shown to be powerful tools for electrochemical hydrogenation [2–21]. A PEM reactor consists of a membrane called a membrane electrode assembly (MEA), which can act as supporting electrolyte, electrode, and heterogeneous catalyst. Therefore, the further addition of a supporting electrolyte is not necessary for the electrochemical reactions using a PEM reactor, which offers clean and environmentally benign organic transformations. Despite these advantages, the utility of PEM reactors in precise organic synthesis has long been unclear. Recently, however, Atobe and co-workers showed that PEM reactors can be used as a powerful and novel tool for precise organic synthesis [22–26]. For instance, they recently reported a stereoselective reduction of alkynes to *Z*-alkenes using a PEM reactor. The use of a Pd/C cathode catalyst and the appropriate cathode potential realize the selective synthesis of *Z*-alkenes [22–24]. They also reported the stereoselective hydrogenation of α,β-unsaturated acids [25] and the reduction of benzoic acids [26].

**Scheme 1:**
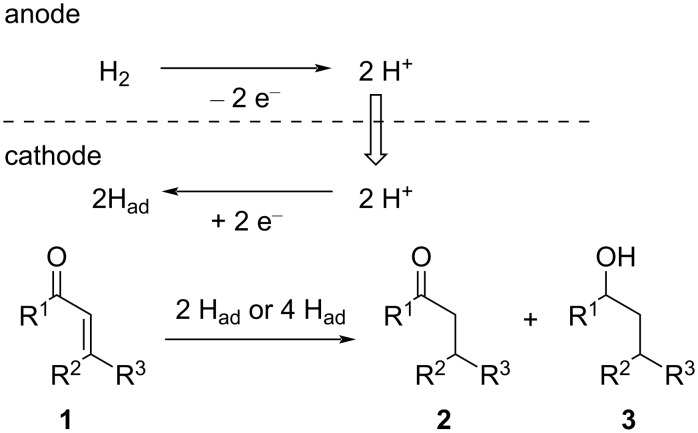
Designed electrochemical hydrogenation of enones **1** with a PEM reactor.

We have been interested in electrochemical transformations for a long time [27–31] and are paying the most attention to the utility of PEM reactors for organic syntheses, especially chemoselective transformations. In our research, we examined the hydrogenation of enones using a PEM reactor. The designed process is illustrated in Scheme 1. Humidified hydrogen gas is passed through the anodic chamber and substrate is passed through the cathodic chamber. The hydrogen molecules are anodically oxidized to two protons. Then, they move to the cathodic chamber and are reduced by the catalyst of the MEA to monoatomic hydrogen species (adsorbed hydrogen, H_ad_) [22]. Thus generated H_ad_ reduces enones **1** to give the corresponding hydrogenated products (ketones **2** and alcohols **3**). The expected advantage of PEM reactors is that the reactivity of H_ad_ should be controllable by the cathode catalyst and electrochemical parameters. Fortunately, we found that chemoselective reduction of enones **1** can be carried out using different cathode catalysts (Pd/C or Ir/C).

## Results and Discussion

### Electroreduction of enones to ketones

**Figure 1:**
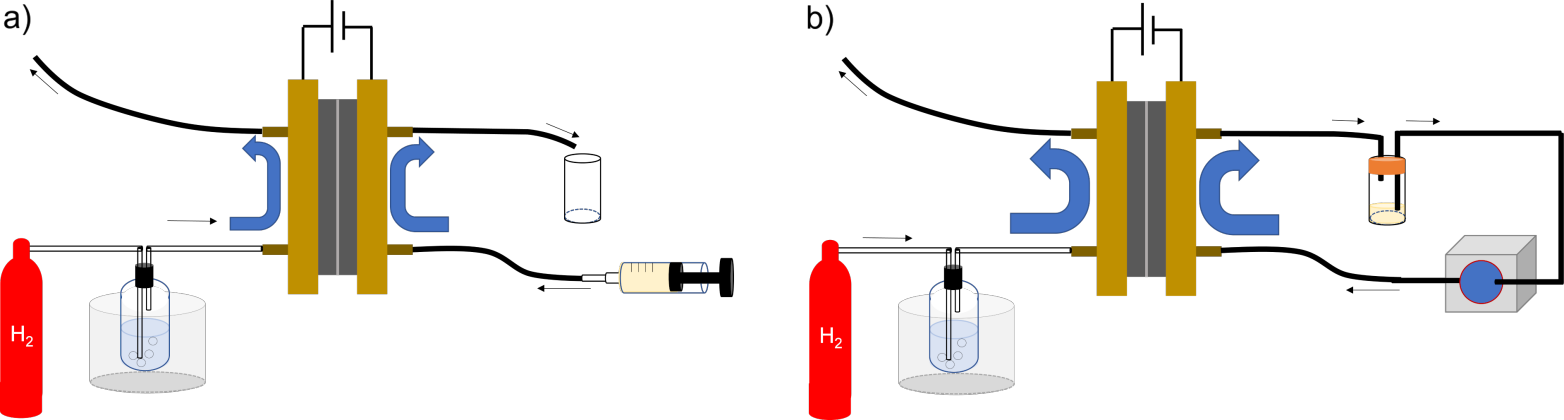
Electrochemical setup of the PEM reactor: a) Electrochemical reduction system with the PEM reactor. b) Circulating electrochemical reduction system with the PEM reactor.

First, we chose cyclohex-2-en-1-one (**1a**) as a model compound, and the electroreduction of **1a** was carried out using a PEM reactor (Figure 1a, a single path). Pd/C was used as a cathode catalyst. Without electricity, trace amounts of cyclohexanone (**2a**) and cyclohexanol (**3a**) were obtained (Table 1, entry 1). With a current of 2.5 mA⋅cm^−1^, **2a** and **3a** were obtained in a yield of 3% (current efficiency 66%) and 0.57% (current efficiency 5.7%), respectively (Table 1, entry 2). While **2a** was obtained with moderate current efficiency, the yield was far from satisfactory. Therefore, electroreduction with a higher current density was examined (Table 1, entries 3–7). The yield of **2a** increased with an increase in the current density (22% yield, 50 mA⋅cm^−1^).

**Table 1:** Effect of the current density on the electrochemical hydrogenation of **1a** with a PEM reactor (a single path).^a^

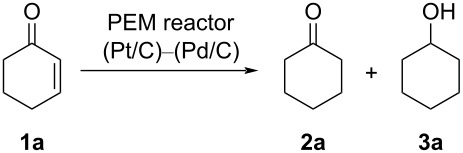					

entry	current density (mA⋅cm^−2^)	conversion (%)	yield (efficiency, %)^b^		selectivity of **2a** (%)

			**2a**	**3a**	

1	0	8	2	0.43	84
2	2.5	<5	3 (66)	0.57 (5.7)	85
3	5	<5	4 (43)	0.52 (2.6)	89
4	10	10	6 (29)	0.52 (1.3)	92
5	12.5	15	7 (27)	0.50 (1.0)	93
6	25	19	12 (23)	0.46 (0.5)	96
7	50	28	22 (23)	0.50 (0.3)	98

^a^Reaction conditions: anode catalyst Pt/C, cathode catalyst Pd/C, concentration of **1a** 1.0 M, solvent dichloromethane, flow rate of the solution of **1a** 0.25 mL⋅min^−1^, flow rate of H_2_ gas 500 mL⋅min^−1^, reaction temperature room temperature. ^b^Determined by GC analysis using *n*-dodecane as an internal standard. Values in parentheses are the current efficiency.

To improve the conversion, we designed a circulating system for the PEM reactor (Figure 1b) and used it for the electroreduction of **1a** (Table 2). First, we carried out the electroreduction of **1a** with a current of 12.5 mA⋅cm^−1^. As expected, **1a** was almost entirely consumed after the passage of 2.0 F⋅mol^−1^, and **2a** was obtained in 67% yield as a major product (Table 2, entry 1). The yield of **2a** and **3a** was almost the same with a current of 25 mA⋅cm^−1^ (Table 2, entry 2). Further, the conversion of **1a** decreased to 82% with a current of 50 mA⋅cm^−1^, but **2a** was obtained in 64% yield with a similar current efficiency (64%). When the reaction was performed in cyclopentyl methyl ether (CPME) as a solvent, the yield of **2a** decreased to 54%, and **3a** was obtained in 11% yield (Table 2, entry 4).

**Table 2:** Effect of the current density and solvent on the electrochemical hydrogenation of **1a** with a circulating PEM reactor.^a^

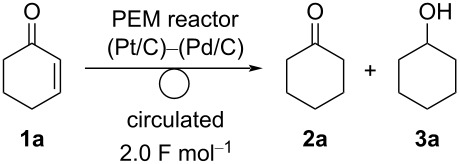					

entry	current density (mA⋅cm^−2^)	conversion (%)	yield (efficiency, %)^b^		selectivity of **2a** (%)

			**2a**	**3a**	

1	12.5	99	67 (67)	5 (11)	93
2	25	96	63 (63)	3 (5)	95
3	50	82	64 (64)	2 (4)	97
4^c^	50	82	54 (54)	11 (22)	83

^a^Reaction conditions: anode catalyst Pt/C, concentration of **1a** 1.0 M, solvent dichloromethane, flow rate of the solution of **1a** 0.25 mL⋅min^−1^, flow rate of H_2_ gas,100 mL⋅min^−1^, reaction temperature room temperature, current density 50 mA⋅cm^−2^. The solution was circulated until the passage of 2.0 F⋅mol^−1^. ^b^Determined by GC analysis using *n*-dodecane as an internal standard. Values in parentheses show the current efficiency. ^c^Performed in CPME instead of dichloromethane.

Next, the effect of cathode catalysts was investigated (Table 3). With Ru catalyst, further reduction of the carbonyl group proceeded, and both **2a** (32% yield) and **3a** (14% yield) were obtained (Table 3, entry 2). With Rh catalyst, the conversion was up to 81%, while the yield of **2a** was similar to that with Ru catalyst (Table 3, entry 3). Similarly, both **2a** and **3a** were obtained with Ir and Pt catalyst (Table 3, entries 4 and 5). In particular, **3a** was obtained preferentially with the Ir catalyst. These results revealed that the cathode catalysts strongly affected the selectivity between **2a** and **3a**. Pd was the best catalyst for the selective synthesis of **2a**, and Ir catalyst should be suitable for the formation of **3a**, regarding the current efficiency and selectivity (Table 3, entry 4).

**Table 3:** Effect of catalysts on the electrochemical hydrogenation of **1a** with a circulating PEM reactor.^a^

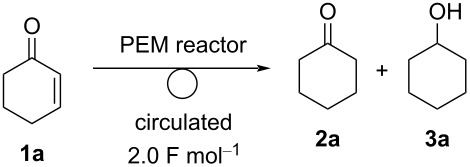					

entry	cathode catalyst	conversion (%)	yield (efficiency, %)^b^		selectivity of **2a** (%)

			**2a**	**3a**	

1	Pd/C	82	64 (64)	2 (4)	97
2	Ru/C	59	32 (32)	14 (28)	70
3	Rh/C	81	37 (37)	19 (39)	66
4	Ir/C	65	24 (24)	26 (52)	48
5	Pt/C	52	11 (12)	27 (56)	29

^a^Reaction conditions: anode catalyst Pt/C, concentration of **1a** 1.0 M, solvent dichloromethane, flow rate of the solution of **1a** 0.25 mL⋅min^−1^, flow rate of H_2_ gas 100 mL⋅min^−1^, reaction temperature room temperature, current density 50 mA⋅cm^−2^. The solution was circulated until the passage of 2.0 F⋅mol^−1^. ^b^Determined by GC analysis using *n*-dodecane as an internal standard. Values in parentheses show the current efficiency.

**Figure 2:**
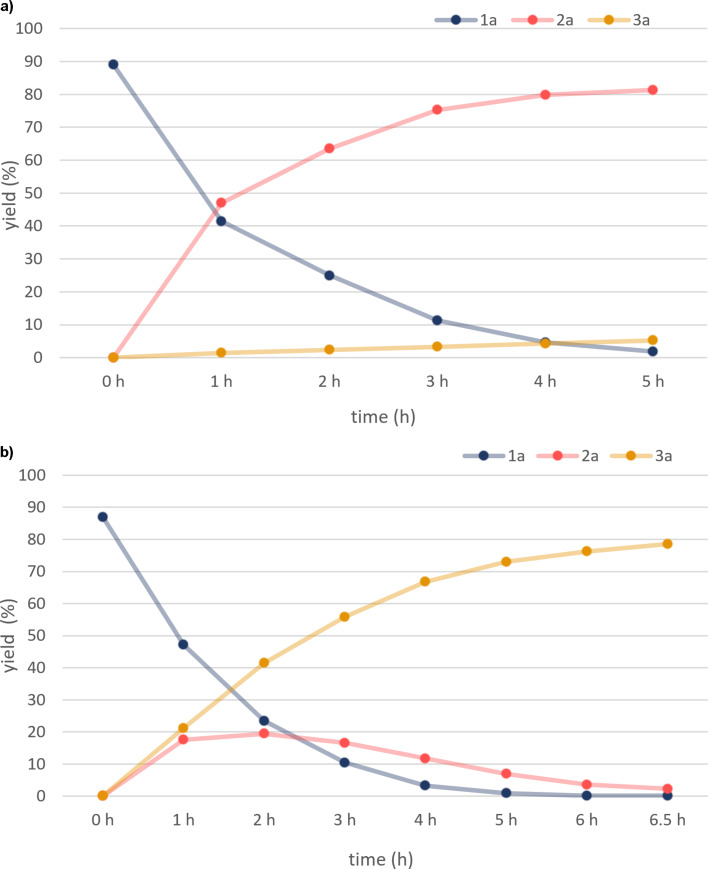
Reaction profile of the electrochemical hydrogenation of **1a** with a PEM reactor using a) Pd/C and b) Ir/C cathode catalyst. The yield of **2a** and **3a** and the recovery of **1a** are shown in red, brown, and blue, respectively.

We also observed reaction profiles of the hydrogenation of **1a** with the use of a Pd/C and Ir/C cathode catalyst, respectively (Figure 2). When a Pd/C catalyst was used, **1a** was hydrogenated to **2a** selectively, and further reduction to **3a** was almost completely suppressed (Figure 2a). In contrast, the use of an Ir/C catalyst afforded both **2a** and **3a**, and generated **2a** was smoothly reduced to **3a** by further electrolysis (Figure 2b).

**Scheme 2:**
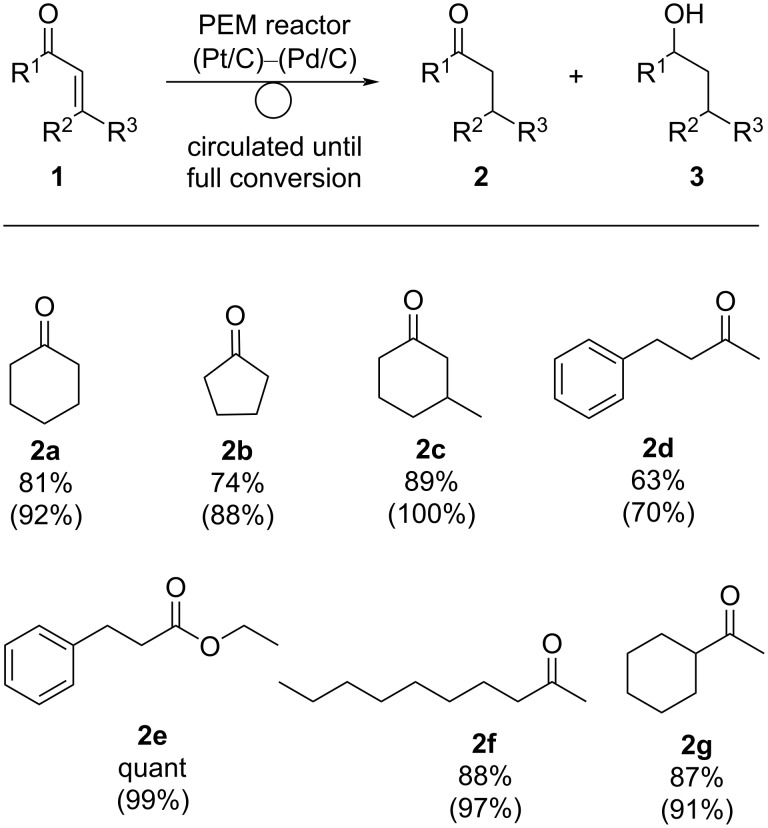
Electrochemical hydrogenation of several enones **1** with a circulating PEM reactor using a Pd/C cathode catalyst. Reaction conditions: anode catalyst Pt/C, cathode catalyst Pd/C, concentration of **1** 1.0 M, solvent dichloromethane, flow rate of the solution of **1** 0.25 mL⋅min^−1^, flow rate of H_2_ gas 100 mL⋅min^−1^, reaction temperature room temperature, current density 50 mA⋅cm^−2^. Charge was passed to the circulated solution until **1** was consumed. The yield was determined by GC analysis using *n*-dodecane as an internal standard. Values in parentheses show the chemoselectivity of **3**, which was calculated as yield of **2** / yield of (**2** + **3**).

As mentioned above, ketone **2a** was obtained selectively with the use of a Pd/C catalyst for the cathode (Table 3, entry 1). To clarify the scope of the reaction, we carried out the electrochemical reduction of several enones **1** using Pd/C cathode catalyst (Scheme 2). After current was passed to the circulating system until **1a** was consumed, the ketone **2a**, obtained by the exclusive reduction of the C=C moiety, was obtained in 81% yield with a chemoselectivity of 92%. Similarly, cyclopentanone **2b** was obtained from the corresponding enone **1b** in 74% yield (88% selectivity). Substituted cyclohexanone such as 3-methylcyclohex-2-en-1-one (**1c**) gave the desired product **2c** selectively in 89% yield (100% selectivity). A benzene-conjugated ketone **1d** and an ester **1e** could also be subjected to electroreduction to afford the corresponding ketones **2d** and **2e** in 63% and quantitative yield, respectively. Linear enone **1f** gave the desired ketone **2f** in high yield (88%, 97% selectivity). Reduction with the PEM reactor also proceeded smoothly with enone **1g**, which has a cyclohexene moiety, to give the corresponding ketone **2g** in 87% yield (91% selectivity). As shown so far, several kinds of enones **1** could be subjected to electroreduction using the PEM reactor to afford ketones in high yield and selectivity.

### Electroreduction of enones to saturated alcohols

**Scheme 3:**
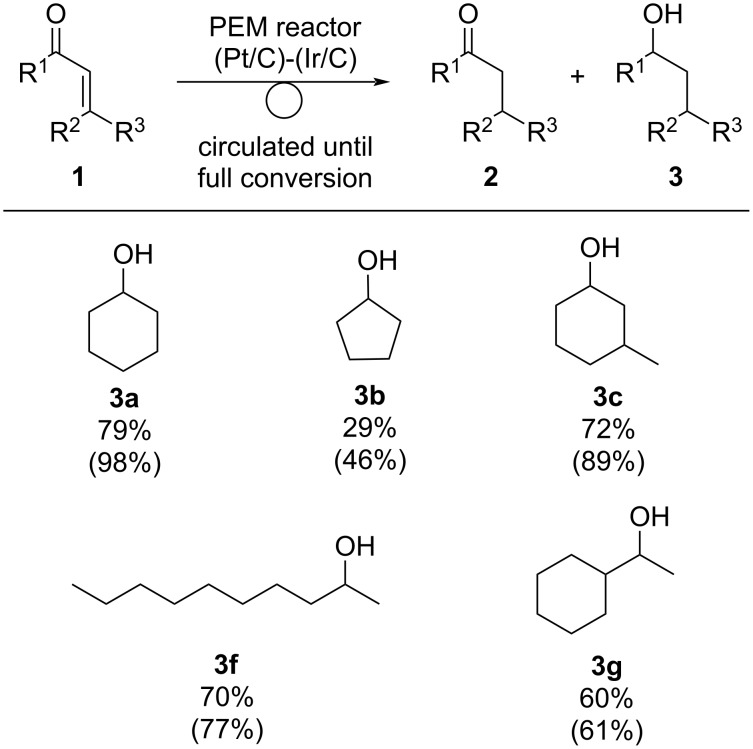
Electrochemical hydrogenation of several enones **1** with a circulating PEM reactor using an Ir/C cathode catalyst. Reaction conditions: anode catalyst Pt/C, cathode catalyst Ir/C, concentration of **1** 1.0 M, solvent dichloromethane, flow rate of the solution of **1** 0.25 mL⋅min^−1^, flow rate of H_2_ gas 100 mL⋅min^−1^, reaction temperature room temperature, current density 50 mA⋅cm^−2^. Charge was passed to the circulated solution until **1** was consumed. The yield was determined by GC analysis using *n*-dodecane as an internal standard. Values in parentheses show the chemoselectivity of **3**, which was calculated as yield of **3** / yield of (**2** + **3**).

We next examined the electrochemical reduction of several enones **1** to saturated alcohols **3** using an Ir/C catalyst for the cathode (Scheme 3). Full conversion of **1a** under the indicated conditions gave **3a** in 79% yield with 98% selectivity. In contrast, electroreduction of cyclopent-2-en-1-one (**1b**) gave cyclopentanol **3b** in 29% yield (46% selectivity), but the reason has not been elaborated yet. With 3-methyl-2-cyclohexen-1-one (**1c**), alcohol **3c** was obtained 72% yield with good selectivity (89%). Both **1f** and **1g** could be used in this reactions, and the corresponding alcohols **3f** and **3g** were obtained as major products.

### Mechanistic studies

**Scheme 4:**
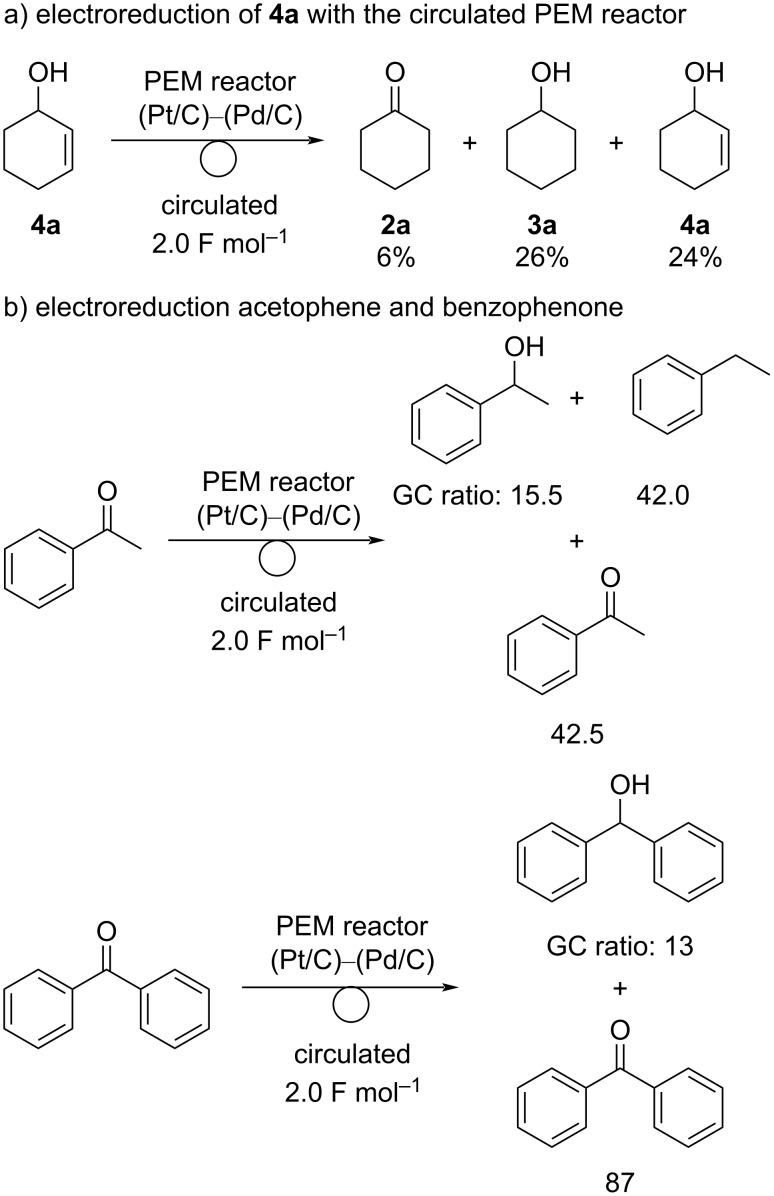
Mechanistic studies.

To gain further insight into the reaction mechanism of the chemoselectivity of a Pd/C cathode system, some additional reactions were carried out (Scheme 4). Electroreduction of **4a** as a starting material was carried out using the circulating PEM reactor equipped with a Pd/C cathode. Compound **4a** has not been observed under the standard reaction conditions performed so far. The reduction of **4a** did not proceed efficiently. Compound **3a** was obtained as a major product (26% yield) and **2a** was obtained in 6% yield (Scheme 4a). Hydrogenation of the alkene moiety of **4a** would proceed selectively, and **2a** would be generated via a transfer hydrogenation reaction from **3a** as a hydrogen donor [32–35]. These results suggest that electroreduction of **1a** would afford **2a** directly and not via **4a**. Electroreduction of acetophenone did not proceed efficiently, and 42.5% (GC ratio) of acetophenone was recovered with ethylbenzene as a major reduced product (Scheme 4b). We assumed that the reduction would proceed via an enol or enolate intermediate. The reduction of benzophenone also did not proceed smoothly, and only 13% of benzophenone was converted. These results suggest that a Pd/C cathode significantly targets an alkene moiety over a carbonyl group, predominantly leading to the reduction of the C=C moiety.

**Scheme 5:**
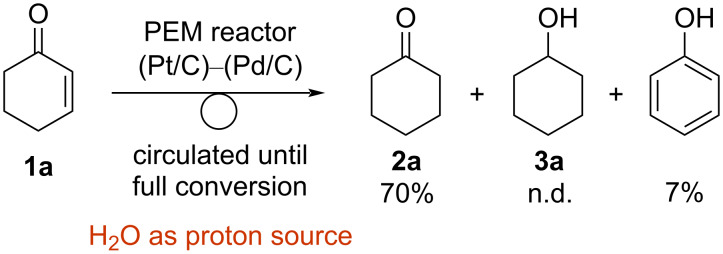
Electroreduction of **1a** with the circulating PEM reactor using H_2_O as a proton source.

Finally, the electroreduction of **1a** was carried out with the use of H_2_O as a proton source by the PEM reactor with a Pd/C cathode catalyst (Scheme 5). Similar to the reaction with H_2_, the electroreduction proceeded with high chemoselectivity, and the desired ketone **2a** was obtained in 70% yield, whereas alcohol **3a** was not observed. Interestingly, the generation of phenol was observed (7% yield), probably because **1a** could serve as a hydrogen donor due to the low concentration of hydrogen [32].

## Conclusion

In conclusion, we have developed a system for the electroreduction of enones using a PEM reactor. The reactions proceeded under mild conditions, and highly chemoselective reductions were achieved with the use of appropriate cathode catalysts.

The use of a Pd/C cathode gave carbonyl compounds selectively. In contrast, saturated alcohols were obtained selectively with an Ir/C cathode. The reaction with H_2_O as a proton source was also achieved. With this reaction system, chemoselective reduction can be performed using only electricity and water, and the product can be easily obtained by simply concentrating the solution coming out of the outlet of the flow system. We are currently trying to reduce various functional groups using this system and shall report the results at a later time.

## Supporting Information

File 1 Experimental details.
