# Supplementary material for: Electrochemical hydrogenation of enones using a proton-exchange membrane reactor: selectivity and utility
Source: Beilstein J Org Chem. 2022 Aug 19;18:1055–61. doi: 10.3762/bjoc.18.107 (PMC9443409; doi:10.3762/bjoc.18.107)
Supplement: File 1 — Experimental details. [file Beilstein_J_Org_Chem-18-1055-s001.pdf]

BEILSTEIN JOURNAL OF ORGANIC CHEMISTRY

Supporting Information

for

Electrochemical hydrogenation of enones using a proton-exchange membrane reactor: selectivity and utility

Koichi Mitsudo, Haruka Inoue, Yuta Niki, Eisuke Sato and Seiji Suga

Beilstein J. Org. Chem. **2022,** *18,* 1055–1061. doi:10.3762/bjoc.18.107

Experimental details

License and Terms: This is a supporting information file under the terms of the Creative Commons Attribution License (https://creativecommons.org/licenses/by/4.0). Please note that the reuse, redistribution and reproduction in particular requires that the author(s) and source are credited and that individual graphics may be subject to special legal provisions.
The license is subject to the *Beilstein Journal of Organic Chemistry* terms and conditions: (https://www.beilstein-journals.org/bjoc/terms)

1. Instrumentation

Gas chromatography (GC) analyses were performed by using a Shimadzu gas chromatograph (GC2014) with a capillary column (thickness of film 0.25 µm, length 30.0 m, inner diameter 0.25 mm, InertCap WAX, GL Sciences, Inc., carrier gas helium). Constant-current electrolysis was carried out with a DC power supply (KIKUSUI PMX350-0.2A).

2. Materials

All chemicals were purchased from commercial suppliers. All chemicals were used without further purification. Dichloromethane was purchased from nacalai tesque, Inc. 2-Cyclohexen-1-one (**1a**) was purchased from Tokyo Chemical Industry Co., Ltd. As for manufacturing the membrane electrode assembly (MEA), Nafion® perfluorinated membrane (Nafion® NRE212) was purchased from Sigma-Aldrich Co. Nafion® perfluorinated resin solution (5 wt % in mixture of lower aliphatic and water, Nafion® DE521) as ionomer solution was purchased from Sigma-Aldrich Co. or Furukawa agency Co., Ltd. Fuel cell catalysts (TEC10E50E; Pt/C, TECRh(ONLY)E30; Rh/C, and TECRu(ONLY)E30; Ru/C were purchased from Tanaka Kikinzoku Kogyo K. K. (TKK). Pd/C and Ir/C were purchased from ISHIFUKU Metal Industry Co., Ltd. Gas diffusion layers (GDL39BC Diffusion Media or GDL39BB Diffusion Media) were purchased from SGL CARBON GmbH.

3. Preparation of MEA

MEA was fabricated with 0.5 $mg \cdot cm^{-2}$ of metal loading amount. Fuel cell catalyst (0.2440 g), deionized water (1.2487 g), Nafion® perfluorinated resin solution, and 1-propanol (1.3874 g) were stirred in a glass vial. This mixture and zirconia balls (diameter 3.0 mm, 10 pieces) were added to a Teflon® vessel. The vessel was placed in a planetary rotation pot mill (PULVERISETTE, Fritsch Japan Co., Ltd.) and rotated at 200 rpm for 20 min. After the rotation, catalyst dispersion was obtained. Gas diffusion electrodes (GDE, 5 cm × 5 cm) were homemade by spraying catalyst inks (Nafion®/catalysts weight ratio of 0.8:1, 1-propanol as dispersant) on commercial GDL39BC or GDL39BB to form a catalyst layer and then drying at 120 °C for 3 min. After spraying, the GDE sheet was cut into 1 cm × 4 cm pieces. Two sheets of this catalyst layer were used for the anode and cathode. Nafion® perfluorinated membrane (3 cm × 10 cm) was put between anode and cathode, and the catalyst sides faced the membrane. Finally, this was hot-pressed (0.4 MPa, 150 °C) for 10 min. Pt was used as an anode catalyst material and Pt, Pd, Ir, Ru, and Rh were used as cathode catalyst materials. The loading amount of each metal catalyst was 0.5 $mg \cdot cm^{-2}$. A photo of thus-obtained MEA is illustrated in Figure S1.

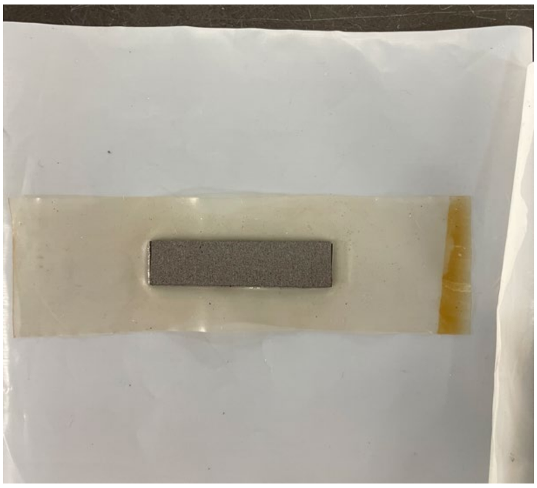

Figure S1: Prepared MEA.

4. Preparation of the PEM reactor

Stainless steel end plates and carbon separators for a PEM reactor were purchased from CHEMIX Co., Ltd. As shown in Figure S2, stainless steel, carbon separators, Teflon® gaskets, and MEA were used to fabricate the PEM reactor, which was tightened to 2.0 Nm with M6 size screws using a torque wrench.

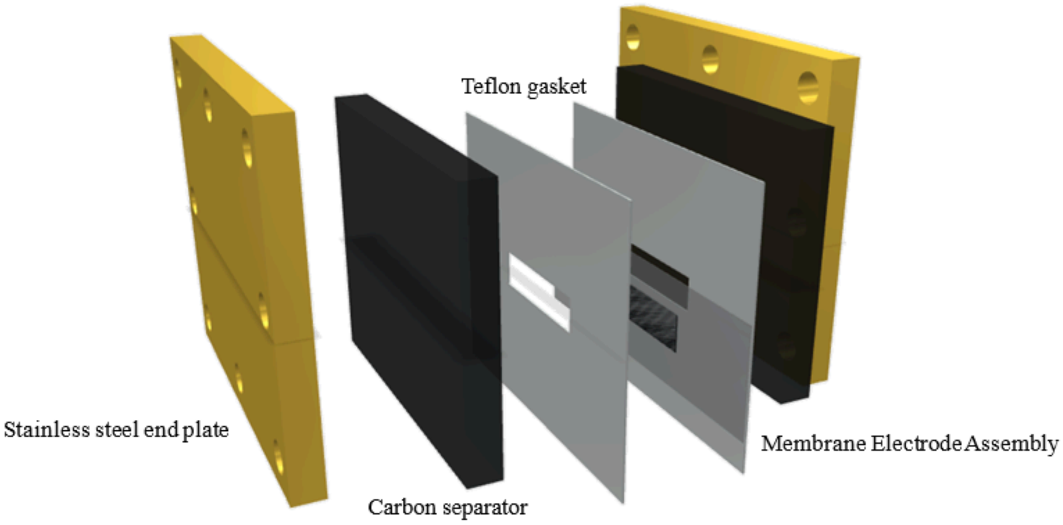

Figure S2: PEM reactor.

5. Hydrogenation of enones with the PEM reactor (single path)

Electrocatalytic hydrogenations were carried out by using the PEM reactor. The MEA (1×4 cm^2 active area) was integrated into the PEM reactor. For the cathodic chamber, enone (1.0 M in CH_2Cl_2) was provided with 0.25 mL·min^{-1} by a single flow system (Figure S3) or a circulating-type flow system (Figure S4). For the anodic chamber, humidified hydrogen gas (flow rate of 100 mL·min^{-1}) was supplied. The obtained reaction solutions were analyzed by gas chromatography (GC2014 with a capillary column, thickness of film 0.25 µm, length 30.0 m, inner diameter 0.25 mm, InertCap WAX, GL Sciences, Inc., carrier gas helium). The products were checked by GC analysis with corresponding standard compounds.

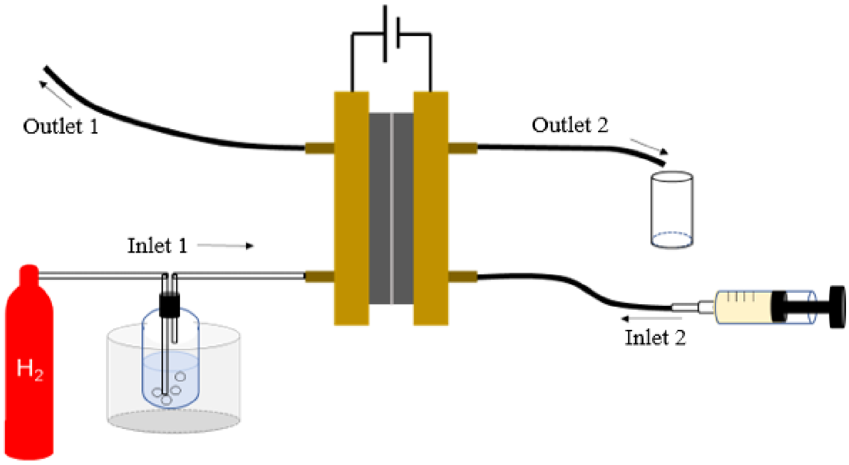

Figure S3: The electrochemical system with a PEM reactor for hydrogenation of enones.

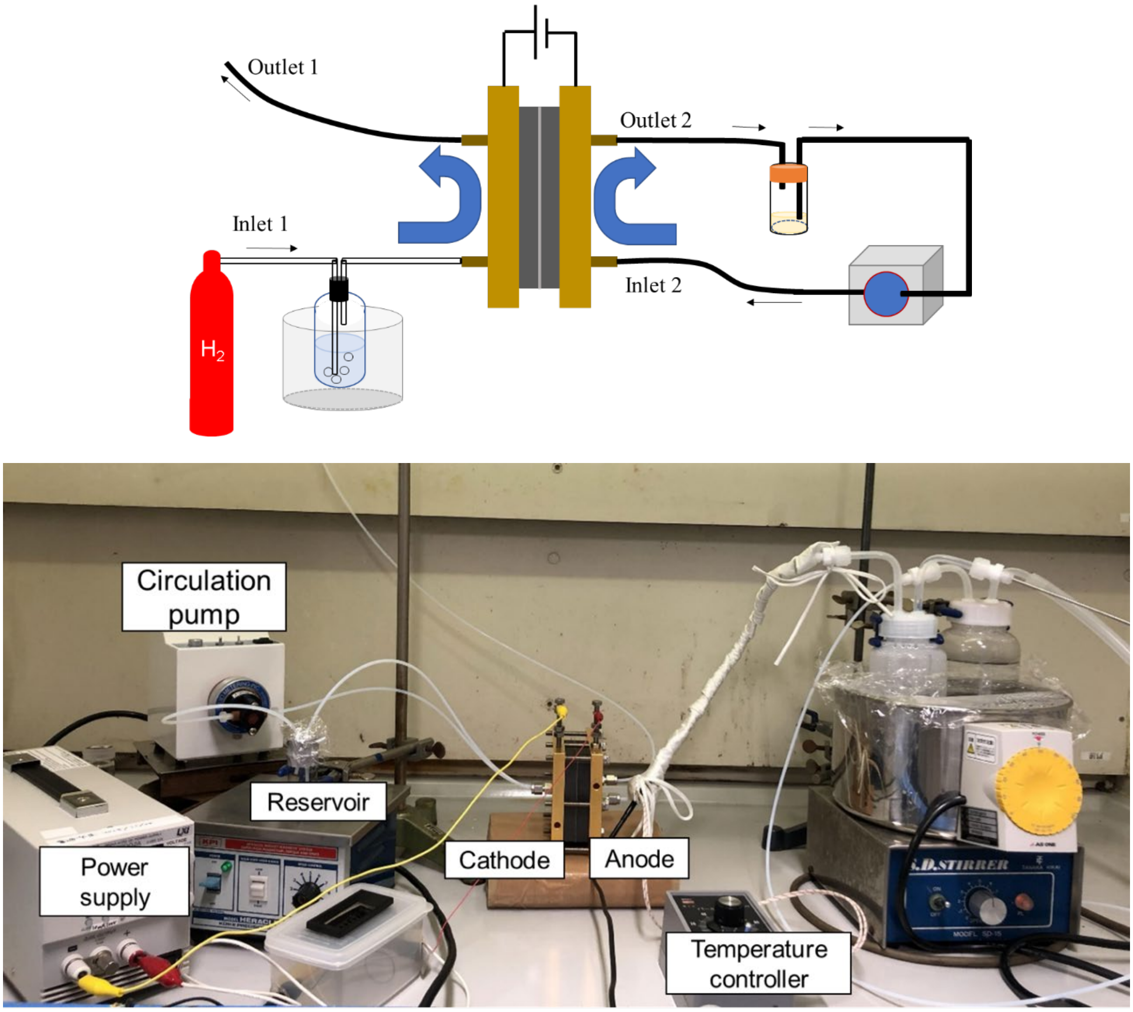

Figure S4: Circulating electrochemical reduction system with a PEM reactor for hydrogenation.
